# Short Term Outcome of Adjunct Foam Sclerotherapy for Varicose Veins in Patients Subjected to RFA at Dhulikhel Hospital, Nepal

**DOI:** 10.1155/2019/4956437

**Published:** 2019-10-07

**Authors:** R. M. Karmacharya, B. Shrestha, A. Singh, N. Chandi, N. Bhandari

**Affiliations:** Department of Surgery (Cardio Thoracic and Vascular), Dhulikhel Hospital, Kathmandu University Hospital, Dhulikhel, Kavre, Nepal

## Abstract

**Background:**

Varicose veins are dilated, tortuous, superficial veins usually seen on lower limbs. Various surgical modalities are available for varicose veins including open surgery (Trendelenburg operation), Endovenous Laser Ablation (EVLA), Radiofrequency Ablation (RFA) and Sclerotherapy. The aim of this study is to look for the outcome of adjunct sclerotherapy for varicose veins done as an adjunct with Radio Frequency Ablation.

**Objective:**

To know the possible outcome regarding benefits and complications of adjunct sclerotherapy with Radio Frequency Ablation.

**Methodology:**

We combined Radio Frequency Ablation of varicose veins with necessary phlebectomy and perforator ligation and performed adjunct sclerotherapy for residual significant varicosities with polidocanol (2%) mixed with 2 ml NS and 2 cc of air (Tessari method) to patients undergoing varicose vein surgery in between 2016 and 2017. Records on complications were enquired immediately following surgery and on 1^st^ follow up done within 3–5 days of the procedure.

**Results:**

Among 256 limbs subjected to varicose veins surgery 51 limbs were given adjunct sclerotherapy. Among them, five limbs had perivenous spillage with some localized swelling while there was allergic reaction in one patient as immediate postprocedural complication. Nine limbs had painful thrombosed veins during early follow-up.

**Conclusions:**

Adjunct sclerotherapy showed complication rate of roughly one tenth and one fifth of the treated cases in immediate and early postoperative follow-up.

## 1. Introduction

Varicose veins are the dilated and tortuous superficial veins characterized by pain, itchiness, heavy sensation, pigmentation and sometimes ulceration [1]. They are part of chronic venous disease spectrum along with telangiectasia and reticular veins, and possess high burden on health care resources [2]. Varicose vein is caused due to venous hypertension, incompetent valves, inflammation, alteration in shear stress, decreased elasticity or structural changes of vein walls causing reflux and pooling of blood in superficial veins [2, 3]. The presence of reflux is determined by clinical examination, handheld doppler or duplex ultrasound [3]. The condition can complicate to venous ulceration if not intervened on time. Cellular level pathophysiology of varicose veins identifies factors as hypoxia, dysregulated apoptosis and changes in the extracellular matrix [4].

There are various modalities of treatment of varicose veins [1, 5]. The modalities include compression stockings, injection sclerotherapy, open surgeries, and minimal invasive surgeries [3]. Minimal invasive surgeries in the form of laser and radiofrequency ablation (RFA) have higher clinical outcomes and patient satisfaction when compared to ligation surgery [5]. During such surgeries, after RFA along with necessary perforator ligation and phlebectomy, if there are significant residual veins then adjunct sclerotherapy is an additional modality that helps to reduce residual varicosities [6, 7]. Foam sclerotherapy has reduced anesthetic use, hospital admission and duration of post treatment bed rest [8]. Concomitant use of foam sclerotherapy and Endovenous Laser ablation (EVLA) have reduced reoperation rates and increased early quality of life [9]. Despite this foam sclerotherapy is known for its complication rate and poor outcomes [10, 11]. Sclerotherapy showed better outcome than surgery on first year following the treatment but after one year following the treatment the outcome was worse which when looked over three to five years were better for surgery [12].

Sclerosing agents damage the endothelial lining and subendothelial collagen fibers are exposed which initiates the coagulation cascade and results in thrombosis of the vessel [13]. Although sclerotherapy was started as liquid sclerotherapy, this has gradually evolved into foam sclerotherapy. European consensus meeting on sclerotherapy has concluded foam sclerotherapy superior over liquid sclerotherapy [14]. Foam sclerotherapy is preferred method due to less side effects and good contact of the drug with the vessel wall [15, 16]. This benefit is more observed when closing larger veins[16]. Depending upon the size of the vein, appropriate volume of foam should be matched [17]. Excess of foam volume might result in migration of foam to deep veins fostering deep vein thrombosis [17]. Foam sclerotherapy is also known to have significant side effects such as thrombotic complications, visual disturbances, neurological complications, dry cough and other rare occurring side effects [18].

Some studies suggest deferring sclerotherapy for 2–4 weeks after RFA as many varicosities diminish in size overtime postprocedure [19]. Adjunct sclerotherapy means the procedure will be done on same anesthesia which will thus be cost effective as no repetition of procedure in the form of sclerotherapy is needed. On the other hand, sclerotherapy has lot of complications. Some of the important complications of Sclerotherapy are superficial vein thrombosis causing pain, pigmentation, allergy, deep vein thrombosis, and embolism. These complications may compel the patient into admission while some like painful thrombosed veins often require re-exploration and excision of the thrombosed superficial veins. In this study we aim to look at the occurrence of complications in patients undergoing adjunct sclerotherapy at Dhulikhel Hospital.

## 2. Methodology

Between January, 2016 and December, 2017 all cases subjected to RFA were combined with necessary phlebectomy and perforator ligation, and for residual significant varicosities adjunct sclerotherapy was performed with polidocanol (2%) mixed with 2 ml NS and 2 cc of air (Tessari method) [20].

We performed RFA using 60 cm or 100 cm VNUS closure fast (Medtronic) RFA catheter under spinal anesthesia. We used 7F needle under ultrasound guidance for cannulation of great saphenous vein. For ultrasound guidance we used Acuson P300 ultrasound (Siemens) with its linear probe (7.5–12 MHz). For site of cannulation, we selected the veins at distal sites that had diameter more than 5 mm, were within 5 mm from skin, has no tributaries within 5 cm of the proximal part and is a straight 5 cm segment in all the feasible cases. RFA generator with heating segment (7 cm long) generating RFA that reached temperature of 120 degree centigrade which maintained for 20 seconds. Two ablation segments were overlapped by 0.5 cm. We performed two segment RFA in the first ablation segment (about 2 cm away from saphenofemoral junction). We did double segment RFA in areas where there were >4 mm perforators or vein diameter was more than 1 cm.

Following RFA for the distal varicose veins, we did necessary phlebectomy in the distal insufficiency point at sites with perforator veins >4 mm. After all the necessary phlebectomy, we assessed the residual varicosities. Adjunct sclerotherapy was done if the residual varicosities had diameter more than 5 mm.

Sclerotherapy was prepared by mixing drug, normal saline and air 2 ml using two 3 mL syringes connected over a three way [21]. We prepared a solution by to and fro flow of the agents which was done for at least nine times and the final solution was injected in the residual varicosities within two minutes under ultrasound guidance (Tessari technique) [20]. Following sclerotherapy, we applied manual compression for 2 minutes at the site of injection. Records on complications were enquired immediately following surgery (within an hour after the procedure) and on 1st follow up (within 3–5 days).

All the cases were subjected for screening Doppler ultrasonography in OPD using the above mentioned Doppler ultrasonography machine in the first follow-up to note if there is any recanalisation in treated vein segment. The recanalisation is noted by presence of both compressibility in the treated vein segment and color flow in color mode.

## 3. Results

Among 256 limbs (144 from males and 112 from females) subjected to varicose vein surgery adjunct sclerotherapy was done in 51 limbs (19.92%). 26 limbs were from male patients and 25 limbs were from female.

In relation to immediate post procedural complication, no complication was noted in 45 limbs (88.23%). In five limbs (9.8%), there was perivenous spillage of sclerosing agent with some localized swelling. In one patient (1.96%), there was allergic reaction which was managed by steroid and antihistamine.

In relation to short term follow-up, no complication was noted in 39 limbs (76.47%). In nine limbs (17.64%), there was painful thrombosed veins. Of them six (11.76%) subsided with analgesics and rest while in three cases, excision of thrombosed superficial vein had to be done. In six limbs (11.76%) there was pigmentation but none of them required any cosmetic intervention. None of the case had ulceration, deep vein thrombosis, infection, embolization. Also, none of the case had significant recanalisation during first follow up.

## 4. Discussion

In our study almost three quarters of the patients didn't have any complications attributed to adjunct sclerotherapy over a period of one week. Although immediate complication rate was only about 12%, this increased over the first follow up. Painful thrombosed veins and pigmentation are the two most common complications.

Ultrasound guided foam sclerotherapy (UGFS) is an important modality of minimal invasive treatment for varicose veins but has some significant side effects. This method has highest chances of recurrence compared to other surgical modalities like stripping, endovenous laser ablation, and radiofrequency ablation [20, 22]. Other notable side effects in UGFS are pigmentation, painful thrombosed veins, allergy to drug [23, 24].

Regarding the biological basis of sclerotherapy, it causes damage to the lining of endothelium initiating coagulation cascade resulting into thrombosis and fibrosis. However, the recanalisation is big issue in UGFS. The rates of recanalization at one year following UGFS has been found to be about 15–20%[22, 25]. One study showed post-procedural partial recanalization rate as 29.1% and complete recanalization as 16% after 6 weeks and even within the cases where complete obliteration had occurred recanalization was present in the quarter of the cases after one year [26]. In a recent meta-analysis comparing outcomes of sclerotherapy and endovenous ablation, the quality of existing evidences comparing effectiveness of ultrasound guided foam sclerotherapy with conventional surgery were found to be low in treatment of short saphenous varicose vein [27]. In a study on recanalisation in two years following ultrasound guided sclerotherapy, over one-third of superficial veins treated with foam sclerotherapy recanalized at one year and about a quarter of superficial veins recanalized at two years [28]. This did not correlate with ulcer recurrence [28].

In order to minimise the risks and decrease the chances of recurrence, adjunct sclerotherapy during surgical procedure is a good option [9, 29]. Some of the immediate complications after foam sclerotherapy for varicose veins are extravasation, pain, visual disturbance, and chest pain while delayed complications include pigmentation, painful thrombosed veins and deep vein thrombosis [30]. In another study, hyperpigmentation was seen in 5.6%, painful thrombosed veins in 3.1% and skin necrosis in 1.8% of cases post sclerotherapy for varicose veins [24].

One of the worrisome complication of sclerotherapy is deep vein thrombosis which occurs in about 1–3%[31, 32]. This complication can be reduced by including the procedure during ablative surgery like radiofrequency ablation [29, 31]. In the study done by Kulkarni et al., it was found that injection into truncal varicosities will have higher chances of DVT in comparison to injection in tributaries [31]. Similarly the recanalisation rate at six months was 8.5% in foam sclerotherapy alone [29]. Although none of the patients in our study had recanalisation, details on the recanalisation can be ascertained in longer follow up. In a study comparing recanalisation rates in first year compared to that in the fourth year, the rates increased rapidly from 4.7% to 28.1% [33].

In our study 88.23% patients were without immediate complication and 76.47% patients were free from short term complication which is similar to other studies [22, 32]. Most common complication noted in our series is painful thrombosed veins noted in nine patients (17.64%). In the study done by Li et al., varicose veins treated by sclerotherapy alone had higher incidence of painful thrombosed veins(14.9%) compared to foam sclerotherapy as adjunct to surgery (1.1%) [29]. Another study showed this as second most common complication occurring in about one-fifth of the cases treated with UGFS [11]. In a review article on complications of sclerotherapy, this occurred in more than 10% of the patients [18].

Some of the strengths of our study are, the study was conducted at a single center, the surgical procedures was done by the same surgical team, and immediate complications and details on the follow up were both noted. However, an important limitation in the study was short follow up duration with no details on the patients after a week. This is important to recanalisation rates as many of the recanalisation happen after a week. Patient's unwillingness to participate in the study after mandatory outpatient consultation is a reason for difficulty to have long term follow up studies. Also there are many possible surgical procedures with variable numbers of perforator ligation, phlebectomy etc., and thus will be difficult to generalise for all the different combinations of the procedures.

Future implication from this study is that adjunct sclerotherapy seems to be a feasible option especially as an add-on treatment modality but it is not devoid of complication limiting its widespread use. More studies on this will be required to answer that for sure as centers where the patients will have hard time following up to their treatment center either due to recurrence from residual varicosities or from complications of adjunct therapy should be weighed in. Hence, we would like to suggest on proper judgment on its selection for appropriate cases.

Trials with higher number of patients and longer follow up duration (more than six months) can be done to have better insight regarding the decision.

## 5. Conclusion

Adjunct sclerotherapy showed complication rate of roughly one tenth of the treated case in immediate postoperative follow-up and one fifth of the treated cases in early postoperative follow-up. Most common complication in adjunct Sclerotherapy was thrombosed superficial veins followed by pigmentation. Recanalisation was not noted in follow-up of a week.

## Data Availability

The data is confidential as per the hospital rules and regulations.
